# Biosurfactant: an emerging tool for the petroleum industries

**DOI:** 10.3389/fmicb.2023.1254557

**Published:** 2023-09-12

**Authors:** Neha Sharma, Meeta Lavania, Banwari Lal

**Affiliations:** Microbial Biotechnology, Environmental and Industrial Biotechnology Division, The Energy and Resources Institute (TERI), New Delhi, India

**Keywords:** biosurfactants, anti-corrosive agent, oil transportation, toxicity, petroleum industries

## Abstract

The petroleum sector is essential to supplying the world’s energy demand, but it also involves numerous environmental problems, such as soil pollution and oil spills. The review explores biosurfactants’ potential as a new tool for the petroleum sector. Comparing biosurfactants to their chemical equivalents reveals several advantages. They are ecologically sustainable solutions since they are renewable, nontoxic, and biodegradable. Biosurfactants are used in a variety of ways in the petroleum sector. They can improve the mobilization and extraction of trapped hydrocarbons during oil recovery procedures. By encouraging the dispersion and solubilization of hydrocarbons, biosurfactants also assist in the cleanup of oil spills and polluted locations by accelerating their breakdown by local microorganisms. The review gives insights into alternative methods for the petroleum industry that are more viable and cost-effective.

## Introduction

**Figure 1:**
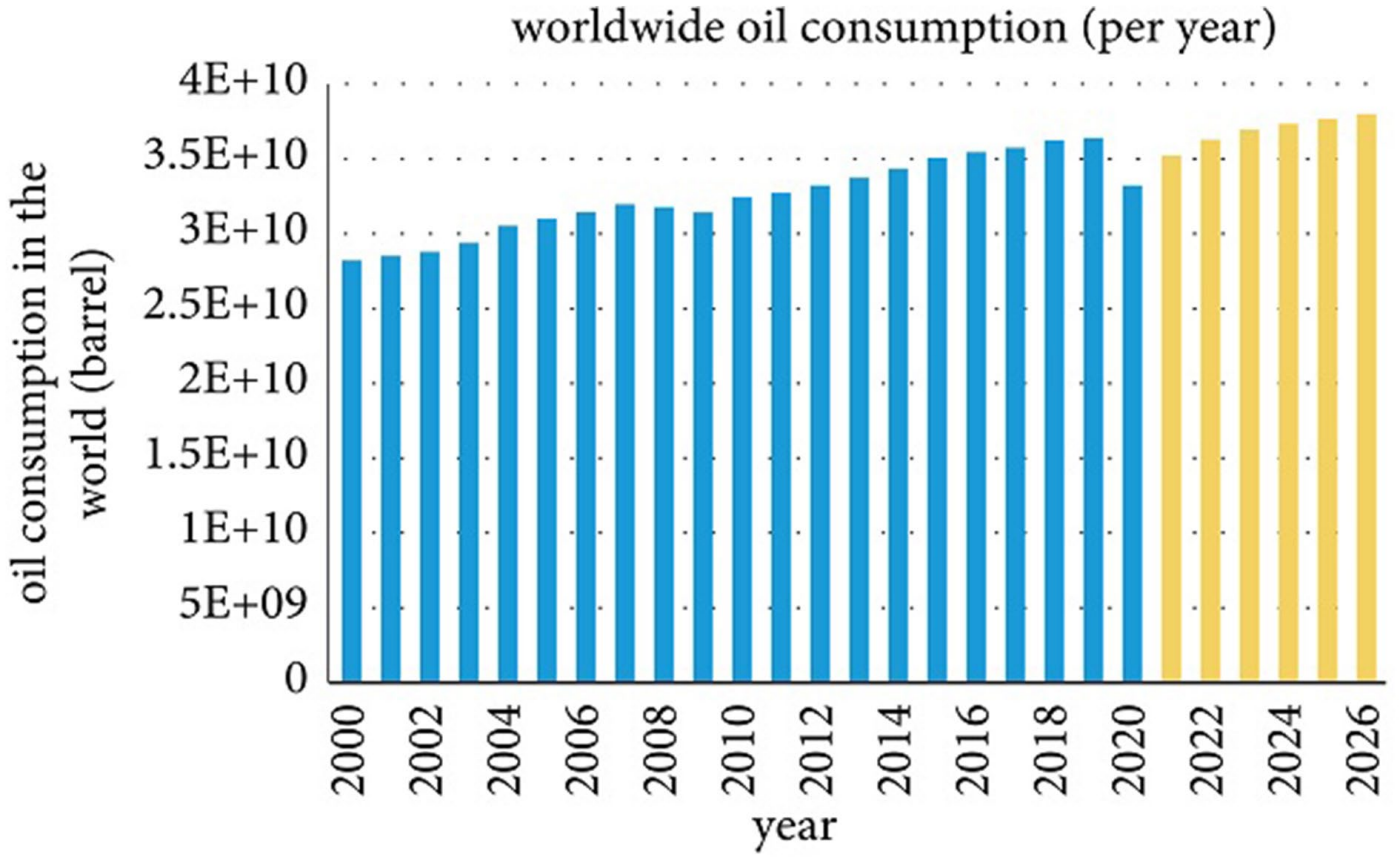
Global consumption of crude oil per year Sonnichsen, 2021.

Today, human life is extremely dependent on fossil fuels and related hydrocarbon products, viz., kerosene, gas, petrol, and diesel (Silva et al., 2014; Geetha et al., 2018). Among the several fossil fuels, crude oil has played an important role in the industrial revolution since the dawn of civilization to secure the world’s energy supplies (Geetha et al., 2018; Bachari et al., 2019). The global oil resource consumption statistics show an increasing trend from the last century to 2019 (36390.5 Mbbl). The primary source of energy is expected to rise sharply in the future, and oil prices will rise (Schulz, 2016). The global crude oil consumption trend from 2000 to the present is increasing as forecasted consumer demand increases in the future (Figure 1). The graph recorded a decline in 2020 due to a global coronavirus pandemic and an extensive shutdown. However, global energy demand is expected to increase in the coming decades (Sonnichsen, 2021).

Around the world, the increasing universal energy demand required a strong energy supply response in addition to limited fossil fuel resources. To regulate the scarcity, researchers are developing techniques to improve the oil recovery from the existing reservoirs. In addition, recognizing alternative resources is paramount to easing the dependence on fossil fuels in the future (Hirasaki et al., 2011; Wei et al., 2014; Ardabili et al., 2019; Nikolova and Gutierrez, 2021). According to the International Energy Agency, oil production is steadily trending toward heavy or ultra-heavy oil production rather than medium to light oil. In countries like China, Canada, Venezuela, Mexico, and the USA, heavy crude oil accounts for about half of the recoverable petroleum resources. Therefore, the development of significant uses for this resource is rapidly becoming an important technology (Ceron-Camacho et al., 2013).

## Emerging technology: petroleum biotechnology

**Figure 2:**
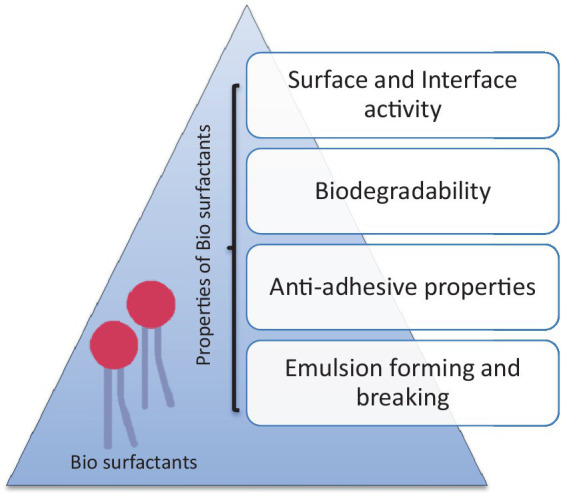
Properties of biosurfactants (Sharma et al., 2022).

Petroleum biotechnology has the aim of introducing biological processes to produce and refine petroleum to generate value-added by-products with minimum pollution output (Silva et al., 2014). The adaptability of microbes, their metabolism, and their intrinsic capacity to facilitate the transformation of complicated raw materials under harsh conditions such as high salinity, temperature, pH values, pressure, and hydrophobicity allow the improvement of those technologies (Montiel et al., 2009). Among various microbial metabolites, the most promising is a biosurfactant in the oil industry (Silva et al., 2014). Biosurfactants are amphiphilic compounds (hydrophilic and hydrophobic domains are present) that are widely known for multiple functions due to their diverse structures and numerous properties, as shown in Figure 2 (Marchant and Banat, 2012).

Various microbial species are capable of producing surface-active molecules to improve the bioavailability of hydrophobic, immiscible, and mostly inaccessible substrates, facilitating enhanced survival under low humidity conditions. The production of biosurfactants generally occurs in miscible hydrophilic and hydrocarbon carbon sources in the medium. The process of synthesis becomes attractive when the researcher develops it from waste substrates (Dziegielewska et al., 2013).

The chemical composition of biosurfactants varies greatly depending on the type of microorganism and can be broadly categorized on the basis of their molecular weight and charge. The biosurfactants are classified into two classes: low molecular weight surfactants (which reduce surface tension between two immiscible liquids) and high molecular weight surfactants (which allow the formation of oil-in-water or water-in-oil emulsions). The high molecular weight surfactants are more complex as they consist of heteropolysaccharides, lipopolysaccharides, lipoproteins, and proteins, whereas the low molecular weight biosurfactants are generally made up of exopolysaccharides that are capable of emulsifying immiscible liquids but are less efficient in reducing the surface tension (Uzoigwe et al., 2015). The list of biosurfactants implemented in the petroleum industry is summarized in Table 1.

**Table 1:** Summary of biosurfactants used in petroleum industries for remediation or enhanced oil recovery.

Biosurfactant type	Microbial species	Application	References
*Low molecular weight biosurfactant*			
Rhamnolipid	*Marinobacter* sp., *Pseudomonas aeruginosa*	Bioremediation, marine oil spill	Tripathi et al. (2019)
*Lipopeptide*			
Surfactin	*Bacillus subtilis*	(Microbial enhanced oil recovery) MEOR	Liu et al. (2015)
Lichenysin	*Bacillus lichenformis*	MEOR	Coronel-Leon et al. (2016)
*High molecular weight biosurfactant*			
Emulsan	*Acinetobacter calcoaceticus*	MEOR	Uzoigwe et al. (2015)
Alasan	*Acinetobacter radioresistens*	MEOR Bioremediation	Toren et al. (2001)
Sphingan	*Sphingomonas* spp.	Oil and gas	Li et al. (2016)
Xanthan gum	*Xanthomonas campestris*	Oil and gas	Kang et al. (2019)

## Application of biosurfactants in petroleum industries

### Enhanced oil recovery (EOR)

Due to the foaming properties of biosurfactants, they can be used in numerous fields, viz., reducing the viscosity of oil and cleaning crude oil tanks as a cleaning agent or detergent. A diverse group of scientists has studied the utilization of biosurfactants in the EOR process. The large scale production of biosurfactants is more expansive than that of chemical surfactants, making them less commercially viable. Biosurfactants are environmentally friendly, less toxic, and can be produced from renewable resources. The use of biosurfactant in EOR can be divided into two heads: *ex situ* (synthesized outside the reservoir) and *in situ* processes (synthesized by indigenous microbes within the reservoir conditions).The laboratory synthesized biosurfactants are directly injected into the reservoir, whereas the *in situ* process identifies the proper microorganisms found in the reservoir that proliferate and synthesize the metabolites such as polymers and surfactants under harsh reservoir conditions (Shahaliyan et al., 2015). Further, after this, the well was shut in and examined for microbial activity and metabolite generation. This has resulted in a significant increase in oil production as a result of the MEOR field trial.

Sharma et al. (2018) studied the potential of thermophilic and anaerobic microbial consortia in incremental oil recovery at 70°C. The consortia were capable of synthesizing biomass, biosurfactants, volatile fatty acids, and biosurfactants under the specially designed nutrient medium. The biosurfactant production by consortia tends to show an effective reduction in permeability at residual oil saturation from 28.3 to 11.3 mD and 19.2% incremental oil recovery in a core flood assay.

McInerney et al. (1990) investigated the production of anaerobic biosurfactants from *Bacillus licheniformis* strain JF2 grown in a medium consisting of glucose mineral salts, NaNO_3_, and yeast extract. When the pH was lowered to 2.0, anionic biosurfactants precipitated from the media, and the surface tension of the media was significantly reduced from 70 mN/m to 28 mN/m. Both JF-2 and surfactin biosurfactant reduced the water surface tension to 27 mN/m. On the contrary, the microbial species *Bacillus subtilis* completely halts the synthesis of surfactin as soon as the hydrocarbon is introduced. Lowering the pH to 2.0 tends to cause the precipitation of surfactins from the *Bacillus subtilis* medium. It can be purified through dichloromethane extraction and acid precipitation.

Bryant et al. (1990) conducted a micromodel to show whether oil can be mobilized through microbial formulations in a porous environment and whether oil mobilization correlates with the recovery of oil in Berea sandstone cores. After the flooding, approximately 60% of the oil was mobilized. Following incubation, the gas bubbles were monitored as well as the emulsification of crude oil. The results showed that gas production, surfactants, and solvents mobilize the oil and lead to a 13% recovery of the oil with the reduced surface tension of the brine.

### Escalated oil transportation through pipelines

**Figure 3:**
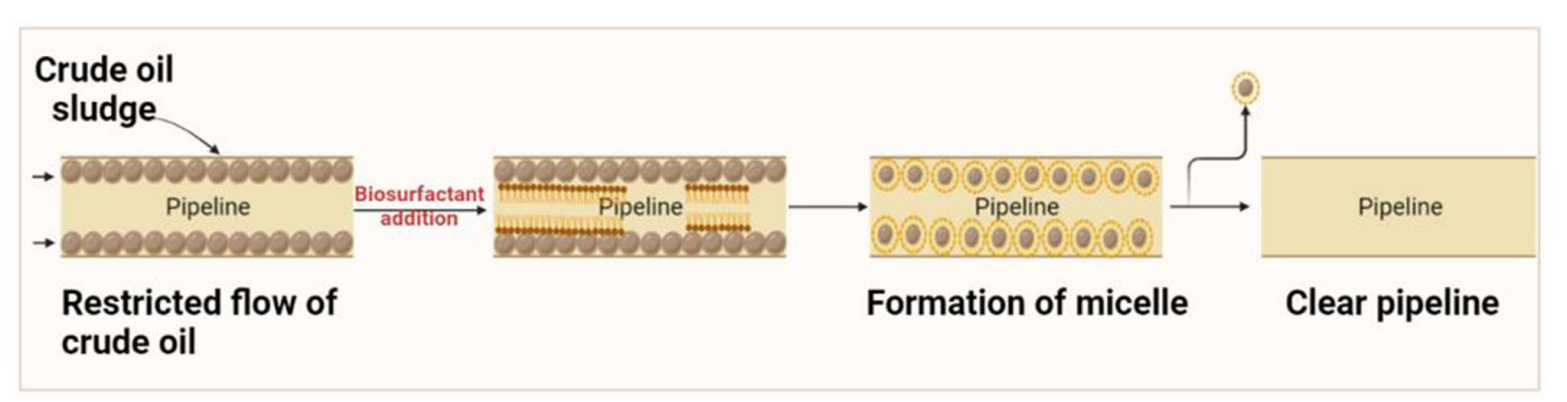
Mechanism of biosurfactant in clearing the pipelines.

Crude oil is generally transported through pipelines from the production sites to the port of shipment or refineries. Such transportation often presents operational challenges that limit economic feasibility, especially for heavy or ultra-heavy crude oil. The high viscosity of crude oil due to the high content of paraffin and asphaltene tends to reduce its flow, often leading to sludge building on the inner walls (Figure 3). Due to these issues, there was a reduction in pressure, and the clogging of pipelines was monitored (Ceron-Camacho et al., 2013).

Traditional methods such as heating and diluting with a solvent (xylene or toluene) were employed to reduce the viscosity and break the semi-solid clogging, which increased production expenses and produced toxic waste but the process is not economical [29]. Recently, promising technologies have been developed for preparing stable oil-in-water emulsions through biosurfactants to promote oil mobility. *Acinetobacter* strains are capable of synthesizing the most potent bioemulsifiers known as emulsans and their analogs. The emulsions can be transported for 26,000 miles and can be utilized directly or treated with specific enzymes (methane monooxygenase, non-heme alkane monooxygenase, alcohol dehydrogenase) that facilitate the breaking of the emulsion before use (Mulligan et al., 2014). A report by Amani and Kariminezhad demonstrated the role of emulsan biosurfactant produced by *Acinetobacter calcoaceticus* PTCC1318 in the removal of crude oil from the stainless steel tubing. Emulsan was recommended as suitable for cleaning pipeline tubing (Amani and Kariminezhad, 2016).

### Oil storage tank cleaning

Enormous quantities of oil are stored in refinery oil tanks and transported for long periods by oil tankers or trucks. Most of these storage tanks are regularly cleaned or maintained, which often increases the problem of hazardous practices or the production of harmful waste material. Also, the sludge deposited on the walls or bottom of the tanks is viscous or semi-solid and cannot be removed with conventional pumps. Removal of this sludge is often done manually or may involve steam or hot water or solvents, these practices is time-consuming, labor intensive, costly and results in a large amount of waste disposal (Matsui et al., 2012).

A field trial conducted by Kuwait Oil Company has shown that biosurfactants can efficiently derive from storage tank cleaning activities. It was done by adding 2 tonnes of rhamnolipid biosurfactant-containing media through energy input to generate a vortex in the tank for 5 days at an ambient temperature of 40–50°C. Promising results were obtained, which showed the effective lifting and mobilization of oil sludge from the base of the tank and its dissolution in the form of emulsions. The process recovered approximately 91% of the hydrocarbons in the sludge, and the value of the crude oil should cover the cost of the cleaning process (Galabova et al., 2014).

### Oil spill treatment

The petroleum industry produces large amounts of petroleum sludge during the processes of oil exploration, storage, transportation, and refining. The disposal of such sludge has always been a major concern for the oil industry. For instance, the annual production of oil sludge in China is estimated to be approximately 1 million metric tonnes, primarily from the oil storage tank cleaning process (Hu et al., 2013). In India, the refining industry produces about 28,000 metric tonnes of oil sludge annually (Liu et al., 2011). Oil sludge is an intricate emulsion of different petroleum hydrocarbons, including solid particles, water, and heavy metals. The treatment method has become a highly sought after technology that has received widespread attention (Joseph and Joseph, 2009).

**Figure 4:**
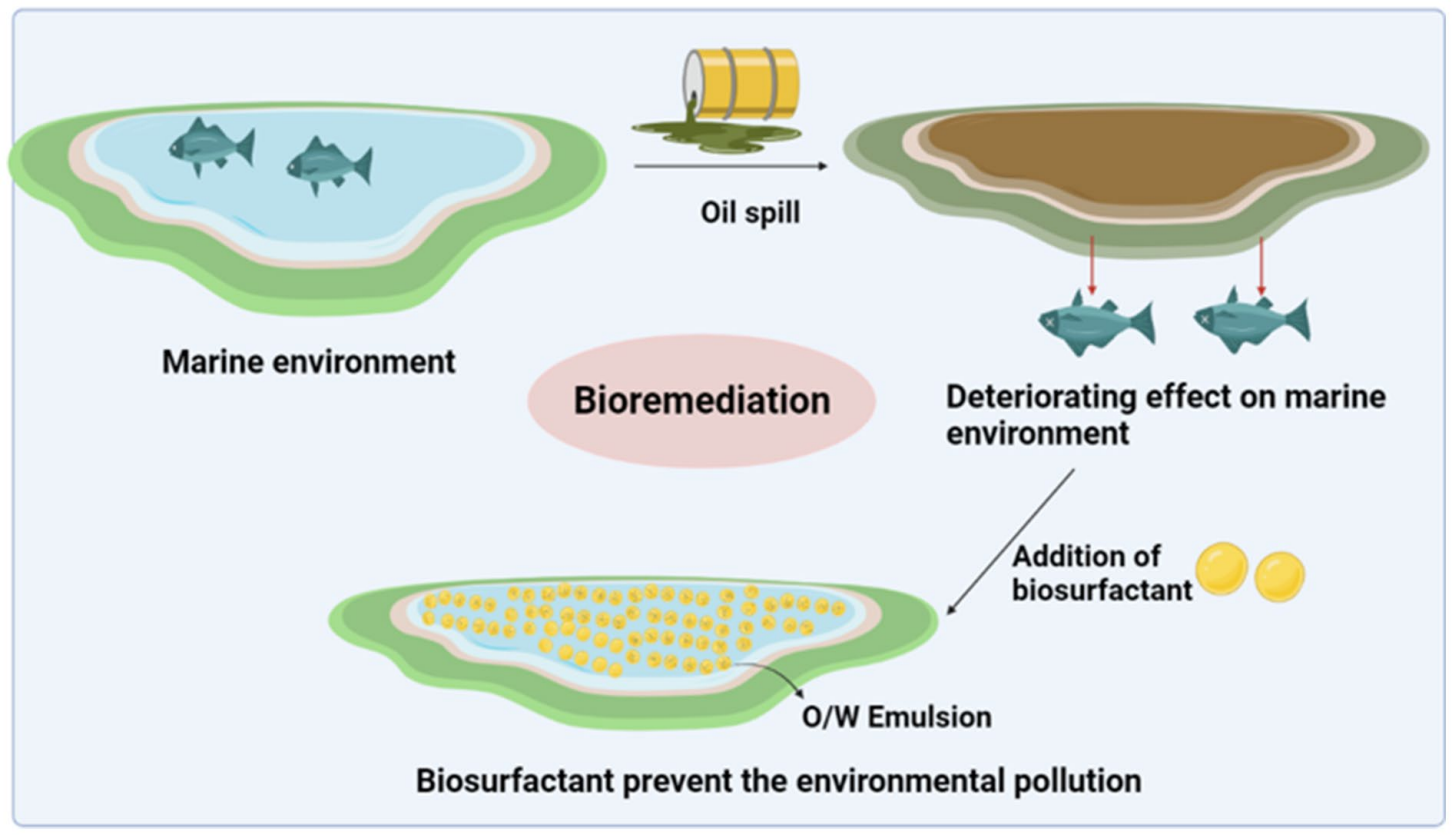
Influence of oil spills on the marine environment and the effective role of biosurfactants in preventing pollution.

A report by Lima et al. investigated the significant removal of oily sludge with the help of biosurfactants produced by five bacterial isolates (*Dietzia maris* sp. LBBMA 191, *Arthrobacter oxydans* LBBMA 201, *Pseudomonas aeruginosa* LBBMA 88A, *Bacillus* sp. LBBMA 111A and *Bacillus subtilis* LBBMA 155) from oil-contaminated sites. Biosurfactants reduce the viscosity and promote the formation of oil–water emulsions, which facilitate mud pumping and emulsion destruction and improve crude oil recovery (Lima et al., 2011). further reduces the amount of sludge by 95%. The separation techniques of centrifugation, ultrafiltration, decantation, flotation, and flocculation are effectively used to separate oil–water mixtures (Painmanakula et al., 2010). Figure 4 demonstrates the impact of spilled oil on the marine environment; the spill drastically affects the entire ecosystem as it contains hazardous components that leak into the system. Biosurfactants can be used to regulate pollution; micelles were formed containing an oil–water emulsion.

### Anti-corrosive agents

One of the major problems in the petroleum industry is corrosion. Entire equipment utilized in oil refineries, storage tanks and transportation pipelines are susceptible to corrosion and negatively impacts the investment in the petroleum sector (NoorEl-Din et al., 2016). Corrosion often begins with the adsorption of protons on the metal surface and the irreversible electrochemical reaction with the metal atoms. There are two possibilities: either the metal cations dissolve in the aqueous phase or they interact with the anions (sulfur). It has been known that such corrosion problems are related to the components of refined crude oil products (Saji, 2010). For prevention, corrosion inhibitors (inorganic or organic chemical surfactants) have been widely studied for many years. The regulation of corrosion in oil fields is a cumbersome process and requires effective inhibitors for applications such as wells, pipelines, storage tanks, etc. (Malik et al., 2011).

### Regulate the proliferation of sulfate-reducing bacteria (SRB)

Anaerobic bacterial groups using an anaerobic bacterium that has sulfate (SO_4_^−^) as a final electron acceptor instead of oxygen are known to cause souring of the oil reservoir and microbial-induced corrosion, making them considered harmful for the oil field (Song et al., 2014). The souring of oil fields occurs because of the H2S and sulfide ions that are produced when the reservoirs are exposed to flooding during the secondary recovery process. The efficiency of secondary oil recovery can be reduced due to the SRB’s biomass and sulfide metal ions (Gaathaug et al., 2014).

To regulate the proliferation of SRBs, biocides such as glutaraldehydes, cocodiamines, and molybdates can be used. Though the economic cost and environmental impact were higher in this case, it tends to generate resistant SRB species against specific biocides that can be lethal in the future. Thus, the cost-effective and environmentally friendly alternative is a biosurfactant that has the potential to regulate the SRB’s growth. Biosurfactants have anti-microbial activity, and their emulsifying activity tends to increase the intracellular osmotic pressure of the cells, leading to the leakage of the entire content (Korenblum et al., 2012). A report by El-Sheshtawy et al. investigated the inhibitory effect of a biosurfactant produced by *Bacillus licheniformis* against the SRBs. The result showed antimicrobial activity against different strains of SRBs and was able to record the complete inhibition of SRBs after 3 h of exposure with 1% crude biosurfactants (El-Sheshtawy et al., 2015).

### Toxicity of biosurfactants during the remediation process

The biosurfactant’s toxicity in the environment is not well defined. Edwards et al. investigated the comparative study of synthetic and microbial surfactants based on toxicity. The results showed that microbial surfactants were less toxic than synthetic surfactants in invertebrate species (Edwards et al., 2003). Though the environmental risks posed by biosurfactants have been assessed by analysing the composition of microbial communities, they have not been adequately evaluated. Biosurfactants have lower toxicity and a higher rate of biodegradability as compared to their counterparts, making them suitable for field applications. The insights of novel biosurfactants need to be carefully examined before releasing them into the environment (Franzetti et al., 2012).

Microbial toxicity of biosurfactants is a potential reason behind the inhibition of the bioremediation process; however, the biosurfactants are non-toxic to microorganisms at concentrations close to CMC values. Another possible cause for the reduced bioremediation rate is the increased toxicity of hydrophobic pollutants due to their increased pseudosolubility. Biosurfactants enhance the aqueous solubility of hydrophobic substrates. Additionally, a few biosurfactants may exhibit selective toxicity to certain pure cultures but show inadequate inhibition against remediation causing microbial populations (Singh et al., 2007). Numerous other chemical agents are used for the remediation process, though these may possess environmental risks and cause a detrimental impact on aquatic organisms (Berninger et al., 2011; Rico-Martinez et al., 2012).

The National Oil and Hazardous Substances Pollution Contingency Plan (NCP) requires proper maintenance as per the U.S. Clean Water Act and Oil Pollution Act to identify the specific products that control oil spills (Hemmer et al., 2011). Products consist of dispersants, surface detergents, surface assemblies, bioremediation agents, and other oil purge control agents. Under NCP, US environmental protection agencies are legally responsible for obtaining toxicity and efficiency benefits from manufacturers before moving dispersants to national product plans (Rico-Martinez et al., 2012).

Probabilistic hazard assessment, including chemical toxicity distribution (CTD), may be useful as the first step to prioritize environmental risks from the use of dispersants. The CTD approach to two acute toxicity records (after NCP-Deep-sided flat lines) includes a median lethal concentration (LD 50) of dispersant alone and dispersants: oil mixture in two standard marine test species, *Menidia beryllina* and *Mysidopsis bahia*. These CTDs recommend that dispersants alone are generally less toxic than oil when compared with dispersant-oil mixtures (Berninger et al., 2011).

Furthermore, numerous tests have been used on various microbial species to assess the toxicity of synthetic and biological surfactants. Lethal concentration (LC50) is a process to estimate the population mortality of a species, indicating that the higher the concentration, the lower the toxicity of surfactant (Rico-Martinez et al., 2012). The germination index (GI) is another way to assess the toxicity of biosurfactants by combining relative vegetable seed germination and relative root elongation. A GI value of 80% was used as an indicator of the disappearance of plant toxicity (Silva et al., 2010; Rufino et al., 2014). Table 2 shows the list of toxicity values of biosurfactants and tested organisms.

**Table 2:** Outcomes of toxicity test of biosurfactant on microbial species.

Biosurfactant	Microbial/vegetables tested species	Toxicity	References
Emulsan	*Mysidopsis bahia* *Menidia beryllina*	LC_50_ (200 mg/L) LC_50_ (300 mg/L)	Edwards et al. (2003)
*Candida sphaerica* UCP 0995 biosurfactant	*Artemia salina* *Brassica oleracea*	LC_50_ (600 mg/L) No toxicity	Sobrinho et al. (2013) Luna et al. (2013)
*Candida lipolytica* UCP 0988 biosurfactant	*Artemia salina* *Brassica oleracea*	No toxicity No toxicity	Rufino et al. (2014)
*Pseudomonas aeruginosa* UCP 0992 biosurfactant	*Artemia salina* *Brassica oleracea*	LC_50_ (525 mg/L) 80%GI	Silva et al. (2010)

GI, germination index; LC50, concentration lethal to 50% of the test species.

## Conclusion

Advances in petroleum biotechnology have become more apparent in recent years, and it has been concluded that many types of biosurfactants are becoming more recognized and valued due to the diversity and efficiency demonstrated in petroleum industry processes. Not only do these compounds play a supporting role, but they are also beginning to play an important role, making them a necessary compound for petroleum biotechnology. The only major advantage of biosurfactants is their biodegradability and non-toxic behavior, which significantly reduce the environmental impact of these compounds compared to chemical surfactants. However, other successful applications are gaining recognition and will lead to increased use in the oil industry.

## Author contributions

NS: data curation, formal analysis, methodology, writing – original draft, writing – review and editing. ML: conceptualization, data curation, formal analysis, funding acquisition, investigation, methodology, project administration, resources, software, supervision, validation. BL: project administration, resources, writing – review and editing.

## Conflict of interest

The authors declare that the research was conducted in the absence of any commercial or financial relationships that could be construed as a potential conflict of interest.

## Publisher’s note

All claims expressed in this article are solely those of the authors and do not necessarily represent those of their affiliated organizations, or those of the publisher, the editors and the reviewers. Any product that may be evaluated in this article, or claim that may be made by its manufacturer, is not guaranteed or endorsed by the publisher.
